# Exploring Trust in Health Insurers: Insights from Enrollees’ Perceptions and Experiences

**DOI:** 10.3390/jmahp13020029

**Published:** 2025-06-09

**Authors:** Frank J. P. van der Hulst, Sanne Huijgen, Anne E. M. Brabers, Judith D. de Jong

**Affiliations:** 1 Nivel, The Netherlands Institute for Health Services Research, P.O. Box 1568, 3500 BN Utrecht, The Netherlandss.huijgen@nivel.nl (S.H.); j.dejong@nivel.nl (J.D.d.J.); 2 CAPHRI, Maastricht University, P.O. Box 616, 6200 MD Maastricht, The Netherlands

**Keywords:** trust, health insurers, managed competition, healthcare system, enrollees, tasks, roles, perceptions, experiences, communication

## Abstract

Managed competition is a key driver in healthcare systems in countries like Germany, Switzerland, and The Netherlands. Trust in health insurers is vital but currently low in The Netherlands. This may be due to perceptions regarding profit motives, negative experiences, media coverage, and a lack of understanding of insurers’ roles. This study explores how enrollees perceive health insurers and how the aforementioned factors contribute to these perceptions. Semi-structured interviews were conducted with 17 participants from the Nivel Dutch Health Care Consumer Panel in March and April 2023. Data were analysed using Braun and Clarke’s six-step method for inductive thematic analysis. Participants generally view health insurers positively in terms of managing finances and ensuring care accessibility. However, some perceive insurers as profit-driven and prioritising cost reduction over individual needs, leading to dissatisfaction. Negative experiences and media coverage also shape these perceptions. Participants believe that insurers should ensure care accessibility and quality, distribute costs fairly, provide guidance, and prioritise preventive measures. To foster trust, insurers should communicate their non-profit status and use of benefits, increase transparency in purchasing decisions, and maintain clear communication about payment obligations. Enhancing communication about their contributions to healthcare and raising awareness of their broader roles may also help build trust.

## 1. Introduction

Managed competition has become a key driver in the healthcare systems of countries like Germany, Switzerland, and The Netherlands. This approach aims to improve care quality and contain costs by fostering competition between third-party purchasers and care providers [1,2,3]. In such systems, health insurers act as purchasers that are responsible for achieving public goals like quality, access to care, and affordability of healthcare services [4,5]. They may accomplish this by negotiating annually with healthcare providers on the basis of volume, quality, and prices. The idea is that when healthcare providers compete with one another to gain the preference of health insurers and health insurers compete to attract enrollees, both parties are motivated to reduce costs and maintain high-quality care. In The Netherlands, managed competition was introduced in 2006 by the Dutch Health Insurance Act (HIA), which assigned insurers the roles of efficient, customer-oriented directors of care. They must control costs, ensure access to care within reasonable time and distance, provide policy information, and mediate to ensure high-quality care [6,7,8,9].

In The Netherlands, health insurers are essentially non-profit organisations, which means that they cannot generate profit for shareholders or private owners [10]. The Dutch healthcare system is based on solidarity and accessibility, with accessibility encompassing aspects such as availability and affordability of care. The system ensures everyone has access to insurance, regardless of their health status or income [11]. Insurers collect premiums from policyholders and are required to use these funds to cover healthcare costs and offer insurance in line with legal guidelines. While health insurers are not focused on maximising profit, they can generate financial surpluses through efficiency or by building reserves for future healthcare needs. However, these surpluses are not distributed to shareholders but can be reserved for financing future healthcare costs or improving service delivery [10].

Each year, health insurers negotiate with healthcare providers to purchase healthcare for their enrollees. Health insurers can negotiate better terms with healthcare providers if they can direct enrollees to preferred providers [1,12,13]. In the Dutch healthcare system, which is based on managed competition, health insurers can use selective contracting. This means that they do not contract all providers, and care from non-contracted providers may not be fully reimbursed. Furthermore, health insurers can also offer healthcare advice to direct enrollees to preferred providers, such as recommending suitable providers, managing waiting lists, and promoting health. Trust in health insurers is crucial for public acceptance and the effectiveness of selective contracting and health advice [14,15]. If these instruments cannot be used to steer enrollees toward preferred providers, this reduces the competition that healthcare providers experience, while competition is what drives the health system to work as intended. When the feeling of competition is absent, health insurers will lose their bargaining position and will be constrained in their ability to steer costs and/or quality of care.

Trust in health insurers in The Netherlands appears to be low. According to earlier research, in 2022, only 26% of respondents indicated that they agree, or completely agree, with the statement that they trust health insurers completely [16]. This is lower than, for example, the level of trust found in 2022 in general practitioners (92%) or hospitals (77%) in The Netherlands [16]. Furthermore, it seems to be lower than the level of trust in health insurers reported in the literature in Switzerland and Germany, which have healthcare systems similar to that of The Netherlands [17,18,19,20]. According to the literature, different factors may contribute to this low level of trust. First, trust in health insurers may be influenced by health insurers being perceived by enrollees as for-profit organisations [19]. This perception can lead enrollees to believe that insurers prioritise profits over patient care. A second factor mentioned in the literature that has a negative impact on trust is prior disputes with health insurers [21]. When enrollees face conflicts or challenges with their insurers, such as reimbursement issues or lack of customer support, their trust in the insurer’s reliability diminishes. A third factor that may affect trust in the health insurer is bad publicity of health insurers in the media [19]. Negative media coverage can shape public opinion and reinforce doubts about the insurer’s integrity and performance. Fourth, little knowledge among enrollees about the role of health insurers in the health care system may be a factor that negatively affects trust in the health insurer [19]. A lack of understanding about how insurers operate and their responsibilities can lead to misconceptions and mistrust.

In this study, we aim to understand the origins of this low trust in health insurers, as trust in health insurers can be linked to their ability to steer enrollees toward healthcare providers [14,15]. As described earlier, this ability is essential for creating competition between health insurers and healthcare providers, which determines whether the system of regulated competition functions as intended. Although the four reasonings for low trust derived from literature seem plausible, there has been little qualitative research on how enrollees perceive health insurers regarding these aforementioned subjects. By examining the enrollees’ perceptions regarding these issues, key elements can be identified that encourage trust among enrollees. This study therefore attempts to gain a more in-depth understanding of how enrollees perceive health insurers, how these perceptions are formed, and how these perceptions may be related to trust. We obtain this understanding based on four research questions that have origins in the four mentioned factors in the literature that are said to play a role in enrollees’ trust in health insurers:

- How do enrollees perceive health insurers in general?
- What are the experiences of enrollees and their relatives with health insurers?
- What is the role of the media in enrollees’ perceptions of health insurers?
- What do enrollees perceive as the tasks and roles of health insurers in the health care system?

This study is particularly relevant for The Netherlands and other countries with healthcare systems based on managed competition, as trust plays a crucial role in ensuring that the system functions as intended. Additionally, for countries with other healthcare systems where health insurers play a role, such as in the private health insurance model in the US [22], this study may offer relevant insights. However, the role of the health insurer in systems other than managed competition may be structured differently or may not exist at all. For instance, in systems that are typically based on the Beveridge model, such as the NHS, where healthcare is funded by the government, consumers do not need to choose between different insurers [22]. In such systems, questions related to trust in the government or the healthcare system in general might be more relevant than those related to trust in health insurers.

## 2. Materials and Methods

### 2.1. Study Design and Participants

To obtain data for this study, semi-structured interviews were conducted. The Nivel Dutch Health Care Consumer Panel (DHCCP) was used to recruit interview participants for this study. The DHCCP’s main goal is to gauge Dutch people’s expectations and experiences with healthcare, as well as their opinions and knowledge about the Dutch healthcare system [23]. The DHCCP is an access panel consisting of about 11,000 people at the moment of the study. They regularly and voluntarily fill out questionnaires on healthcare. Information about several background characteristics of the panel members is available.

Membership to the panel is by invitation only. Individuals cannot join on their own initiative. Upon joining, panel members are informed about the panel’s purpose, scope, methods, and usage. Based on this information, participants can consent to participate. Written informed consent, which has been allowed to be given digitally since 2020, is obtained when a new member registers to the panel. Pseudonymised data are analysed and processed in accordance with the privacy policy of the Dutch Healthcare Consumer Panel. The panel adheres to the General Data Protection Regulation (GDPR) [23]. According to Dutch legislation, approval from a medical ethics committee is not required for research conducted using the panel [24]. Participation is voluntary, and members are not obligated to participate in surveys or answer specific questions. They can terminate their membership at any time without providing a reason. Returning the completed questionnaire is considered consent to participate. Panel members are informed about the questionnaire’s subject and length in the invitation letter.

In February 2023, a sample of 1500 panel members received the annual questionnaire regarding health insurance. The sample was chosen according to criteria that reflect the demographic composition of the Dutch population aged 18 and older in terms of sex and age. Of the respondents in this questionnaire who indicated that they were willing to be contacted for an interview (n = 183), seventeen participants were selected and invited for interviews. The sample was selected based on respondents’ level of trust in health insurers, with eleven participants chosen who reported (very) low trust and six participants selected who reported (very) high trust. Seventeen participants were invited for interviews. In the last interviews, it appeared that no new information or themes emerged, indicating that data saturation was reached. All participants were informed in writing and verbally prior to the interview about the goal and scope of the interview and gave consent to participate. After the interview, the data were pseudonymised, analysed, and processed in accordance with the DHCCP’s privacy policy and the GDPR.

### 2.2. Data Collection

The first author (F.J.P.v.d.H.) conducted all seventeen interviews during March and April 2023. The interviewer had no personal or professional connection with any of the participants. A topic list that included open-ended questions relevant to the four research questions of the study was used to guide the interview process (see Appendix A, Table A1). The questions in this topic list were based on the four previously mentioned concepts from the literature that are said to play a role in enrollees’ trust in health insurers. Together, three of the authors (F.J.P.v.d.H., A.E.M.B., and J.D.d.J.) collaborated to develop the topic list. All the researchers involved are employed at Nivel. The interviews took thirty to forty-five minutes on average. The interviews were conducted via video call using Microsoft Teams or over the phone, based on each participant’s preference. With the participants’ consent, audio recordings of every interview were made for the purpose of verbatim transcription.

### 2.3. Data Analysis

**Figure A1:**
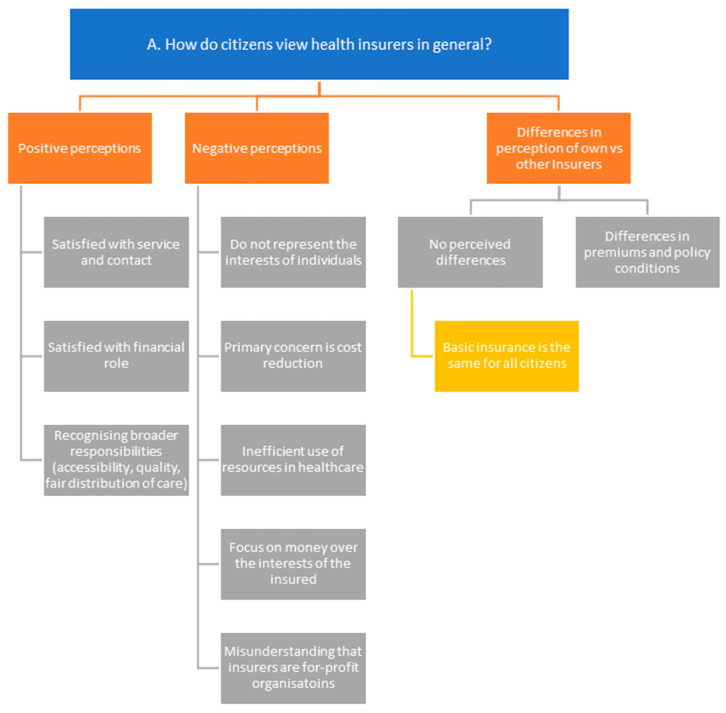
Coding tree related to research question 1: “How do citizens view health insurers in general?”.

**Figure A2:**
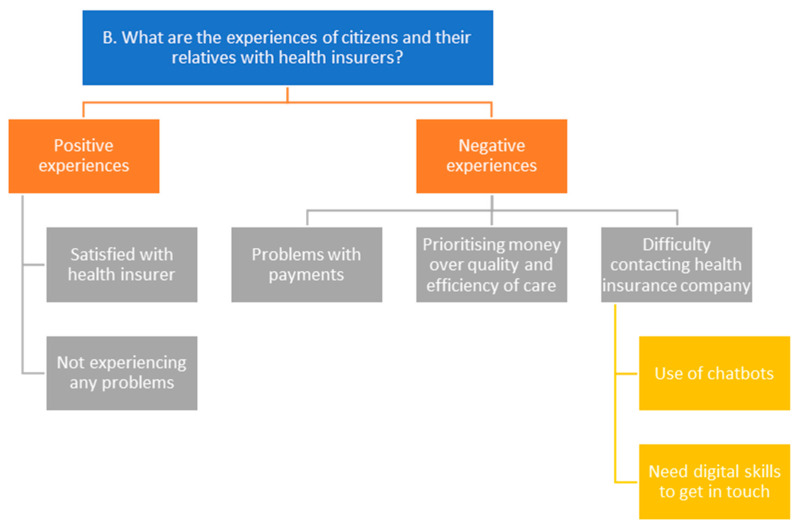
Coding tree related to research question 2: “What are the experiences of citizens and their relatives with health insurers?”.

**Figure A3:**
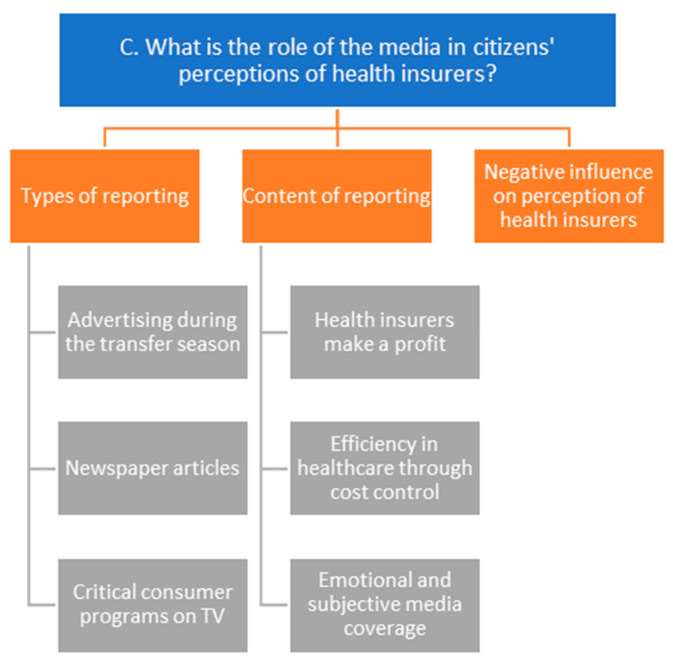
Coding tree related to research question 3: “What is the role of the media in citizens’ perceptions of health insurers?”.

**Figure A4:**
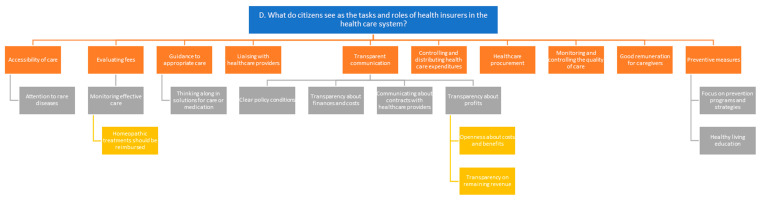
Coding tree related to research question 4: “What do citizens see as the tasks and roles of health insurers in the health care system?”.

Braun and Clarke’s six-step method for inductive thematic data analysis was used to derive themes from the data [25,26]. The first step consisted of familiarisation with the data. After the completion of all the interviews, the audio recordings underwent verbatim transcription and pseudonymisation. To become comfortable with the data, the researchers reviewed all the interview transcripts. Then, in the second step, the researchers created codes to capture interesting data (open coding). One researcher (F.J.P.v.d.H.) coded the first four interviews, while another researcher (S.H.) coded the remaining thirteen interviews. The first two interviews coded by S.H. were discussed between F.J.P.v.d.H. and S.H. In addition, to increase the reliability of the study, a third researcher (A.E.M.B.) coded four interviews independently [27]. The differences in assigned codes were debated by F.J.P.v.d.H., S.H., and A.E.M.B. Subsequently, in the third step, the researchers searched for themes. A coding tree (axial coding) was constructed according to the predetermined themes based on the research questions (see Appendix B, Figure A1, Figure A2, Figure A3 and Figure A4). During the fourth step, the researchers refined and reviewed the themes. By methodically perusing the coded transcripts, it was possible to validate the predetermined themes. Eventually, the constructed coding tree was evaluated by different members of the research team to increase the reliability of the themes that were investigated in connection to the data (selective coding) [27]. In the fifth step, the themes were defined and named. Thereafter, distinct summaries of the results of the interviews were written to give a thorough rundown of the research. The sixth and last step, writing the report, included gathering the themes and summaries into a comprehensive overview of all the findings. In addition, a “peer debriefing” technique was implemented to strengthen the study’s credibility [27]. In this step, a panel of peer researchers who were not involved in the study met to discuss the draft article. A few changes were made to the draft version based on this peer feedback. The interviews were coded using the MAXQDA software (Release 22.1.1).

## 3. Results

### 3.1. Participants

The interviews were completed by all seventeen participants. The gender distribution, with eight male and nine female participants, was nearly equal. According to the background information about the Nivel Dutch Health Care Consumer Panel, the youngest participant was 31 years old, the oldest was 81 years old, and the average age of the participants was 51 years old. Seven participants were highly educated (professional higher education or university), eight had a medium level of education (secondary or vocational education), and one had a low level of education (none, primary school or pre-vocational education). For one participant, no data were available. Eleven participants reported having one or more long-term or chronic conditions, while six participants reported having no long-term or chronic conditions. There was one participant with a low income (less than EUR 1750 net per month), eleven with a medium income (between EUR 1750 and 2700 net per month), and five with a high income (more than EUR 2700 net per month).

### 3.2. Interview Results

In this section, we answer the four research questions one by one based on the results of the interviews. A schematic overview of the results of the interviews (a coding tree) can be found in Figure A1, Figure A2, Figure A3 and Figure A4 in Appendix B.

#### 3.2.1. How Do Citizens View Health Insurers in General?

In general, the participants’ perceptions of health insurers are favourable. Most participants are satisfied with the service and contact provided by the health insurer, such as support with healthcare claims or telephone assistance and answering questions. The interviews revealed that a number of participants perceive the health insurer purely as a financial handler and are satisfied with how this role is performed. Other participants recognise that the responsibilities of the health insurer are broad, including roles such as guaranteeing good accessibility and quality of care or ensuring the equitable distribution of healthcare costs.

However, participants’ perceptions about health insurers are not only positive. For instance, several participants feel that health insurers do not represent the interests of the individual. They perceive health insurers as being overly concerned with financial matters. They feel that the focus of health insurers is primarily on reducing costs and believe that more attention should be given to the individual circumstances of their enrollees. Regarding these individual circumstances, one of the participants made the following statement:

*“I also find the human aspect important. You know, that individual circumstances are taken into account. Because people are biological and biology is not an exact science, sometimes there are situations that fall outside the norm. And it’s nice to put everything into the system, but sometimes there’s no box that person fits into, you know, and then you have to tweak things a bit, and I find that willingness to do so important as well.”*

Participant #3

One participant believes that the focus on money is especially noticeable regarding the issue of medication shortages, as indicated by the following statement:

*“I think they have far too much power and that it’s all about money. I also believe that their privatisation, which happened years ago, was a bad decision (…) It’s really all about money. And people who need medication always have to... Well, at the moment, it’s a hot issue with medications being unavailable. People suffer too much from this. I think it’s not focused on care, which is what it should be about.”*

Participant #12

A few participants indicated that health insurers’ focus on reducing costs sometimes also comes at the expense of the efficient use of resources in care. One participant, for example, mentioned having difficulty obtaining sufficient incontinence supplies for her father because the health insurer only allowed two diapers per day, which was insufficient. The participant said that after much effort, this was increased to eight diapers per day, which was too many. This led to frustration for the participant over the rigid rules of the health insurer and the impression that resources in healthcare could be used more efficiently.

Several participants have an inaccurate perception that health insurers in The Netherlands are allowed to make a profit and distribute it to shareholders. One of the insured commented on this belief as follows:

*“Operationally, they do good work and facilitate the flow of money. I don’t have much trust in their interventions. Look, they are ultimately all companies that need to make a profit and have shareholders, and I think that shareholders are prioritised over patients.”*

Participant #15

Others indicated that they do not know whether health insurers are allowed to make a profit, as follows:

*“I’ve never wondered about it. It seemed so illogical to me that they would make a profit, that I’ve never questioned it. But do they?”*

Participant #11

With regard to differences in view of their own health insurer versus other health insurers, most participants do not view them very differently. Some of these participants indicated only perceiving differences in premiums and policy conditions between health insurers. However, the fact that basic insurance is the same for all citizens in The Netherlands makes the differences small, according to some of them.

#### 3.2.2. What Are the Experiences of Citizens and Their Relatives with Health Insurers?

Participants have varying experiences with health insurers, including personal experiences and experiences heard from relatives or friends. Some participants indicated that they like their health insurer and that they are satisfied with their health insurance company because they have not experienced any problems with them. Other participants shared examples of negative experiences. An example of a negative experience is that payments due to the mandatory deductible in The Netherlands are an issue. A participant mentioned, for example, that a relative’s unexpectedly high medical expenses resulted in problems, as follows:

*“My brother-in-law was once picked up by ambulance somewhere and taken to a hospital not here in our area, but to another hospital because there was no room here. (…) Later he got a bill and that was because he did have a higher deductible I think. And that bill from the ambulance then had to be paid in stages because they didn’t have more money in their bank account. And then I think that just shouldn’t be allowed.”*

Participant #14

Additionally, some participants indicated that in their experience, the health insurer prioritises money over quality and efficiency of care. One of the participants gave an example involving the price of prescribed drugs. She indicated that she has observed that medications are currently being phased out and replaced with cheaper options that do not always work or are not appropriate for people at all because of side effects, as follows:

*“My mother had medication for blood pressure, which worked perfectly without side effects, and all of a sudden it wasn’t allowed to be given anymore. And then I asked the pharmacist, ‘Why not?’. And so it was because 0.01 per cent of a cent per pill was more expensive than the alternative that she would get side effects from. But they get them because those 0.01 percentage points are important. And then you have more side effects, but you have a cheap drug, so yes, that creates aversion.”*

Participant #3

During the interviews, it emerged that not all participants found that it was simple to contact their health insurer. One participant mentioned that they faced waiting times on the phone before reaching their health insurer, depending on the time they called. Furthermore, another participant expressed dissatisfaction with her experience with health insurers using chatbots for customer service, presumably to save costs. In addition, some participants indicated that an increasing number of digital skills are needed to get in touch with health insurers, such as the need to use a digital identification system used in The Netherlands that allows residents to securely access government services online (called DigiD), as follows:

*“Well, I have to say, my health insurance company does everything via e-mail. (…) That’s going to be a problem. At one point you could only log in with your DigID and I didn’t have a DigID. And then you had to log in with a smartphone. I don’t have a smartphone, I don’t have a cell phone, I’m one of those people who doesn’t have a cell phone.”*

Participant #3

#### 3.2.3. What Is the Role of the Media in Citizens’ Perceptions of Health Insurers?

Participants indicated that they had seen different types of reporting about health insurers in the media. Participants mentioned that they had seen health insurers’ advertisements, especially at the end of the year during the period between mid-November and December 31, when enrollees can switch health insurers. A number of participants reported seeing newspaper reports and critical consumer programmes on TV. According to one participant, the media frequently brings up the fact that health insurers generate profits. Another participant reported seeing items in the media that address the role health insurers play in achieving efficiency in health care by controlling costs, as shown in the following statement:

*“Then I see particularly the items about to what extent the health insurance companies are interfering with the efficiency of care, the cost of certain drugs and whether or not to use certain drugs and the trade-offs they have to make between care that is used very infrequently versus very widely used care and how those costs should be settled.”*

Participant #1

Several participants indicated that they are aware that the media is often not objective and that media coverage is often emotionally driven. This emotional reaction towards the health insurer can be both positive and negative. According to one participant, large health insurers receive the majority of the media attention, with smaller health insurers receiving less attention overall. Some participants expressed their opinions regarding health insurers portrayed in television shows. One of the participants made the following statement:

*“Or things that have unfortunately gone less well or positive, yes. Often the negatives are expressed anyway. That’s good for the audience ratings, though.”*

Participant #10

Another participant made the following statement:

*“It’s often negative. But I also think because that’s kind of what’s out there. But there’s also a fallacy in that because everything that stands out comes out in the news. So if a hundred things go right, you don’t hear about it. Does one thing go wrong? That makes the news.”*

Participant #2

Some participants indicated that their perception of health insurance companies is negatively influenced by the media, as follows:

*“Yes, my perception does get negatively affected by that, and that’s mainly because then they kind of take it easy off. And what I say is that I think yes, they really only look at those costs and how they can keep the price as low as possible. And that sometimes comes at the expense of patient care, so to speak.”*

Participant #7

#### 3.2.4. What Do Citizens See as the Tasks and Roles of Health Insurers in the Health Care System?

Participants were asked what they see as tasks of the health insurer in the healthcare system. This led to different answers. Some participants indicated that it is the health insurers’ duty to ensure the accessibility of care. According to these participants, health insurers have a responsibility to purchase care and to guarantee that care is distributed fairly. One of the participants made the following statement:

*“I do expect that they ensure these negotiations go well so that everyone can access care everywhere. And I believe that is not always the case now, that you can really go everywhere, but rather to a large extent. But yes, I would expect them to make care as accessible as possible for people. So that you have the choice to go somewhere yourself.”*

Participant #7

With regard to the accessibility of care, another participant made the following statement:

*“Well, ensure that the patient can make a choice from the treatment options that are available. So options of hospitals nearby or far away.”*

Participant #9

One of the participants specifically mentioned that attention should also be paid to the accessibility of care for rare diseases, as follows:

*“I also expect that they enforce a certain level of care, meaning that they say [to healthcare providers] ‘you must also provide this care’, even if it yields little benefit for the caregivers themselves in that regard. Focusing more on rare diseases in particular.”*

Participant #1

Furthermore, some participants think that insurers should play a role in critically evaluating reimbursements. According to several participants, the health insurer should more closely monitor what constitutes truly effective care. Some participants mentioned that homeopathic treatments are not covered by health insurers, while certain treatments prove to be effective for some patients. One participant made the following statement about this issue:

*“Well, I heard this week that homeopathic remedies don’t work at all. But why do they still exist? Look at what does work and maybe promote that, because there are definitely things that are effective. I think that through natural medicine there are enough options to solve problems. Unfortunately, this is not always reimbursed, and that’s a shame.”*

Participant #16

In addition to the accessibility of care, some participants also consider guidance on suitable care to be an important role. They expect the health insurer to identify solutions for issues related to care or medication supply and to maintain contact with all healthcare providers. A few participants also see facilitating accessible and personal contact as an important role for health insurers. They expect the health insurer to provide customised services in which the interests of the individual patient are represented. A participant made the following statement:

*“I do think it’s very important that, even though everything is digital nowadays, insurers remain reachable by phone. It’s crucial that everyone can easily contact their insurer if there are things that need to be discussed.”*

Participant #6

Transparent communication regarding policy conditions was also mentioned by some participants as an important role. Participants indicated that policy conditions are not always clearly formulated and that there is a lack of transparency regarding the financial aspects and costs of care. Furthermore, they consider it important for the health insurer to communicate which providers they have and have not entered into contracts with. One participant made the following statement:

*“Well, I think it’s very important that they [health insurers] are very transparent about this [contracted healthcare providers]. Actually, every year I find it strange that you hear and see on the site that it is not yet known whether we will have a contract with them next year. Surely that should just be known the moment we get the choice to choose a health insurer.”*

Participant #8

Furthermore, some participants believe that it is important for the health insurer to be transparent about any profits it may make, as well as how any remaining revenue is used. Some of the participants suspect that the premium is spent on organisational costs, including advertising. They believe that the costs and benefits of the health insurer should be openly disclosed. One participant who was not aware of what health insurers do with profits made the following statement:

*“No, but I do think it’s important that those, that they provide that. Because ehm, actually you should just know what your health insurer does with its profits of course.”*

Participant #14

According to some of the participants, health insurers also play an important role in controlling and distributing healthcare spending. These participants indicated that health insurers have a role to play in healthcare purchasing, making difficult decisions about healthcare spending, and controlling healthcare costs. One participant pointed out that it is important for the health insurer to keep income and expenses balanced, as follows:

*“The main task is to manage and disburse the pot of insurance money according to the clients’ or patients’ demand for care. Then. The main part of that task is balancing the incoming flows and the outgoing flows and they can turn to that by increasing premiums on the one hand or keeping healthcare costs in check on the other.”*

Participant #1

In addition to distributing healthcare spending, one participant considers it a duty of the health insurer to monitor and steer the quality and efficiency of care. In addition, a few participants believe that it is critical for health insurers to maintain high standards of care, compensate healthcare workers well, and prepare for the future (sustainable care). According to one participant, health insurers should make investments in the medical field to prepare the Dutch healthcare system for the future, as follows:

*“Should you not put that [care premium] somewhere else, for instance into making more staff available in the end? Because we do face such a problem together. An ageing population in The Netherlands. More and more people are getting diseases and therefore more demand for care. And I do wonder whether the health insurers are sufficiently aware that this demand for care will soon have to be met. So the quality is good now. But what about ten years from now? I’m curious about that.”*

Participant #7

Several participants believe that health insurers must prioritise preventive measures more. When putting prevention programs and strategies into place, health insurers ought to take the initiative more often, according to these participants. One of the participants indicated that health insurers should take a leading role in promoting healthy living among citizens and expressed the following opinion:

*“They should try that, thus with education. They are already working on that. Towards healthier living of human beings. And they have to take the lead in that even more. But they don’t have to do that alone, but they can initiate that. Yes, they have to finance it, so I also think they should have a leading role in that.”*

Participant #15

## 4. Discussion

The aim of this study was to gain an in-depth understanding of how enrollees perceive health insurers, what factors contribute to these perceptions, and how these perceptions may be related to trust.

First of all, according to the literature, trust in health insurers may be influenced by health insurers being perceived by enrollees as for-profit organisations [19]. Our study indicates that some participants believe that health insurers prioritise money over the quality and efficiency of care. This is in line with results from previous research, which has shown that over two-thirds of enrollees think that health insurers consider saving money more important than purchasing the care they need [28]. Additionally, some participants feel that health insurers are mainly focused on profit maximisation instead of delivering quality care. The impression that financial considerations outweigh patients’ health and well-being may potentially lead to a feeling of alienation and mistrust, as enrollees may not feel supported in their search for the best care. Dutch health insurers are not allowed to distribute profits, and there are rules governing their reserves [10]. This did not appear to be known to all participants. Several participants mistakenly believed that health insurers in The Netherlands are allowed to make a profit and distribute it to shareholders. Health insurers could therefore focus more on communicating that they are organised as not-for-profit cooperatives, as well as disclosing what they do with their revenue. Furthermore, it is essential to explore how negative perceptions can be changed by, for example, providing more transparency regarding the decisions made by health insurers in terms of quality and costs when purchasing care. Our results and recommendations are similar to those of the study by Yildirim et al., which also noted that the majority of participants view health insurers as profit-driven organisations and recommended that more attention should be given to addressing the misconceptions surrounding this perception [29].

Secondly, experiences with the health insurers are mentioned in the literature as one of the factors that contribute to trust in health insurers [19]. Therefore, we investigated our participants’ experiences with health insurers and where they see room for improvement. Some participants reported unexpected payments due to the mandatory deductible as a negative experience with health insurers. The experience of having to pay may undermine trust, as this can create a financial burden for individuals, especially those with lower incomes or chronic health conditions that require frequent medical visits. This financial strain may lead to feelings of frustration and resentment towards the insurer. It is important for the health insurer to be transparent about payments due to the deductible, as a lack of clarity around payments may undermine trust in health insurers [28].

Another issue participants reported from their experience was the contact options provided by the health insurer. Some participants mentioned long waiting times on the phone. Furthermore, a participant mentioned the use of chatbots, which create a sense of distance and impersonality. The fact that enrollees generally do not favour this mode of communication is in line with previous studies, which found that insurance enrollees typically tend to reject interacting with chatbots [30,31,32,33,34]. Additionally, some participants find that an increasing number of digital skills is required to contact health insurers, posing an extra barrier. This finding shows how important it is for health insurers to offer personal contact options.

A third factor that is mentioned in the literature and may contribute to trust in health insurers is media coverage [19]. The results of our study reveal that according to the participants, the media often highlights profitability and cost control with respect to health insurers, evoking mixed reactions. While some participants critically assess media coverage and are aware of its emotionally charged nature, others report a negative influence on their perception of health insurers. The literature shows that public trust is closely linked to communication about the factors that create value for stakeholders [35]. Positive reporting on how health insurers are committed to issues that create value for healthcare and society, such as quality of care, accessibility, patient satisfaction, innovation, prevention, and cooperation with healthcare providers, may contribute to a more positive perception. This may translate into greater public trust [35]. In addition, umbrella organisations of healthcare providers and patient associations may be strong allies in regaining public trust [36]. If they openly express support for purchasing plans from health insurers, it is likely that their members will also support these plans [36].

Lastly, a lack of knowledge among enrollees about the role of health insurers in the health care system has been identified in the literature as negatively affecting trust in the health insurer [19]. We therefore examined what roles participants attribute to health insurers. Participants expect health insurers to make care accessible and fairly distributed, closely monitor what constitutes truly effective care, provide guidance on appropriate care, and ensure transparent communication about policy conditions and healthcare costs. Additionally, they emphasise the importance of preventive measures and proactive initiatives in promoting public health. The variety of expectations surrounding the tasks and roles of health insurers mentioned by participants show that there is knowledge among enrollees about the different roles of health insurers in the healthcare system. However, not all enrollees recognise the roles of the health insurer in their entirety, and not all expected roles align with the actual roles, leading to false expectations. For instance, some participants believe that health insurers decide what is included in the basic health insurance package, noting that homeopathic treatments are not covered. However, in The Netherlands, the National Health Care Institute (Zorginstituut Nederland) actually makes these decisions and advises the Minister of Health, Welfare, and Sport (VWS), while health insurers are responsible for implementing and adhering to these decisions. Research by Yildirim et al. shows similar results regarding the extent to which policyholders in The Netherlands are fully aware of the purchasing role of the health insurer [29]. To increase knowledge, health insurers could highlight their role in purchasing care, ensuring access to care, setting premiums, verifying claims, and promoting preventive and sustainable care in communications, thereby fostering a full understanding and appreciation of their services. Furthermore, communicating about the execution of their broad role allows enrollees to see the execution of important tasks reflected. This is important because previous research has shown that enrollees who feel that health insurers do not perform important tasks have less trust in health insurers [28].

We recommend clear communication about health insurers’ broad role, non-profit status, decision-making regarding quality and costs when purchasing care, and enrollees’ payment obligations, as this may improve trust in health insurers; however, it is important to note that the effectiveness of communication may also depend on the recipients’ health insurance literacy and the communication channels used [37,38,39,40,41]. Additionally, not everyone may be sufficiently interested in information about health insurers to actually engage with it. Health insurers and the government should therefore tailor their communication about health insurers to different target groups and ensure that this information is accessible through various channels, as this may improve citizens’ attitudes and engagement [42,43].

### Strengths and Limitations

A strength of this study is that it is one of the first to qualitatively examine, through interviews, the factors that the literature associates with enrollees’ trust in health insurers. In addition, a notable strength of this study is that by using the Nivel DHCCP, we were able to approach citizens with a diversity of background characteristics, since this information is available for the panel members. By conducting the interviews not only via Teams but also by phone, less digitally skilled participants were also able to participate. Nevertheless, a limitation is that the Nivel DHCCP lacks people with low literacy skills or people with a migrant background who do not speak Dutch. As a result, these groups were not represented in this study. To include them in the research, we would need to recruit in a different way, for example through snowball sampling or indigenous field worker sampling [44]. Another limitation is that although participants were partly selected based on their reported level of trust in the questionnaire they had completed, this did not always fully align with their interview responses. Initially, we mainly aimed to select participants with low trust to understand their reasons. However, the interviews revealed more variation in trust levels than expected. Although the questionnaire led us to select eleven participants with low trust and six with high trust, the interviews showed five with low trust, six with high trust, and six who were neutral. While this shift was not intended, it may have provided us with a broader and more nuanced understanding of the factors contributing to trust or distrust in health insurers, enriching our results with diverse perspectives as we spoke to different groups represented in society. In addition, while the final group was diverse in gender, age, income, and health status, the respondents were all Nivel DHCCP panel members. These panel members may have an above-average interest in healthcare, leading to a potential bias. Furthermore, certain groups, such as participants with low education, participants with low literacy, and migrants, were underrepresented. Although data saturation was reached, it is possible that if we interviewed people from these groups, additional perspectives might have emerged. Future research could focus on perspectives from these specific groups. Finally, the findings of our study are oriented on the Dutch situation and therefore might not be entirely applicable to other countries’ healthcare systems. While the results should be interpreted within the context of the Dutch healthcare system, findings such as the importance of transparency are also relevant in the international context.

## 5. Conclusions

This study highlights the diverse perceptions of health insurers among enrollees. While some view insurers primarily as financial managers, others acknowledge their broader role in ensuring accessibility, quality of care, and equitable cost distribution. The literature indicates that trust in health insurers in The Netherlands is generally low. To improve perceptions and foster trust, insurers could enhance communication about their non-profit status and the use of profits. Increasing transparency about decision-making regarding the quality and cost of care could also help change negative perceptions, as previous studies have suggested that transparency can enhance trust [29,45].

Negative experiences, such as unexpected payments and impersonal contact methods, may undermine trust. Therefore, clear communication about payment obligations and maintaining personal contact options are important for improving trust. The media’s potential influence suggests the need for strategic communication about insurers’ contributions to healthcare, highlighting their role and impact positively. Lastly, increasing awareness through transparent communication about the health insurers’ broad role and activities can help build trust. Further research could explore effective communication strategies to enhance transparency towards enrollees.

## Data Availability

The minimal anonymised data set is available upon request from Judith D. de Jong (j.dejong@nivel.nl), who is the project leader of the Dutch Health Care Consumer Panel, or the secretary of this panel (consumentenpanel@nivel.nl). The Dutch Health Care Panel has a program committee that supervises the processing of the data of the Dutch Health Care Consumer Panel and makes decisions about the use of the data. This program committee consists of representatives from the Dutch Ministry of Health, Welfare and Sport, the Health Care Inspectorate, Zorgverzekeraars Nederland (the Association of Health Care Insurers in The Netherlands), the National Health Care Institute, the Federation of Patients and Consumer Organisations in The Netherlands, the Dutch Healthcare Authority, and the Dutch Consumers Association. All research conducted within the Consumer Panel has to be approved by this program committee. The committee assesses whether a specific study fits within the aim of the Consumer Panel, which is to strengthen the position of the healthcare user.

## Appendix A

**Table A1:** Interview guide.

Interview Guide Question	Relevant Research Question
1. What can you tell me about how you view health insurers? a. Do you view your own health insurer differently compared to other health insurers? Why or why not? Can you explain?	A. How do enrollees perceive health insurers in general?
2. What can you tell me about your personal experiences with health insurers?	B. What are the experiences of enrollees and their relatives with health insurers?
3. Do people in your area ever share their experiences with health insurers with you? a. If so, what kind of experiences do people around you share?	B. What are the experiences of enrollees and their relatives with health insurers?
4. Do you ever read media reports about health insurers? a. If so, what type of messages do you see in the media?	C. What is the role of the media in enrollees’ perceptions of health insurers?
5. How much trust do you have in health insurers? a. Can you explain that?	A. How do enrollees perceive health insurers in general?
6. What do you expect from a health insurer? a. What do you think the role of health insurers is in the healthcare system? Why? b. What do you think are important tasks for health insurers? Why?	D. What do enrollees perceive as the tasks and roles of health insurers in the health care system?
7. What is your opinion about how health insurers carry out their tasks? Why?	D. What do enrollees perceive as the tasks and roles of health insurers in the health care system?
8. What do you think health insurers can do better or differently?	D. What do enrollees perceive as the tasks and roles of health insurers in the health care system?
9. Do you think health insurers should be allowed to make a profit? a. What do you know about that? b. If so, what do you think about that?	A. How do enrollees perceive health insurers in general?
10. What do you think about the fact that most health insurers are associations or cooperatives, which are non-profit organisations?	A. How do enrollees perceive health insurers in general?
